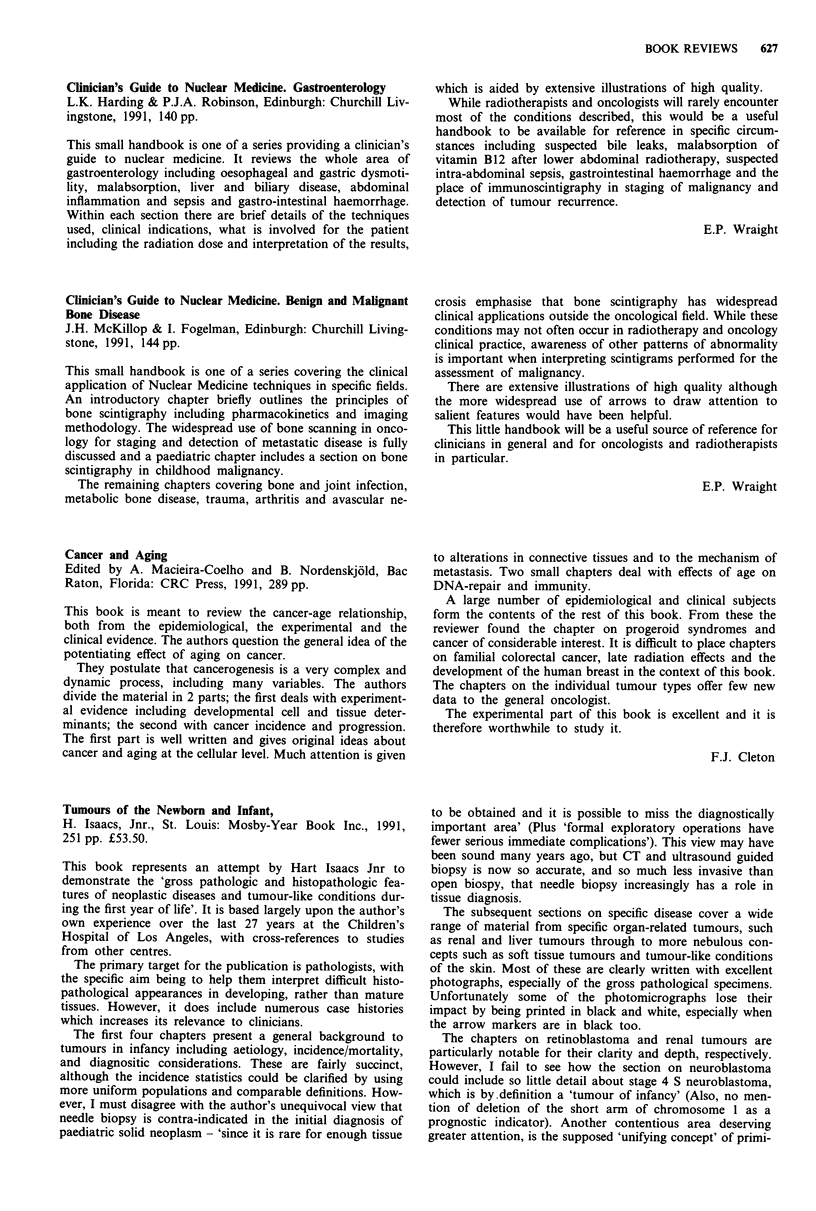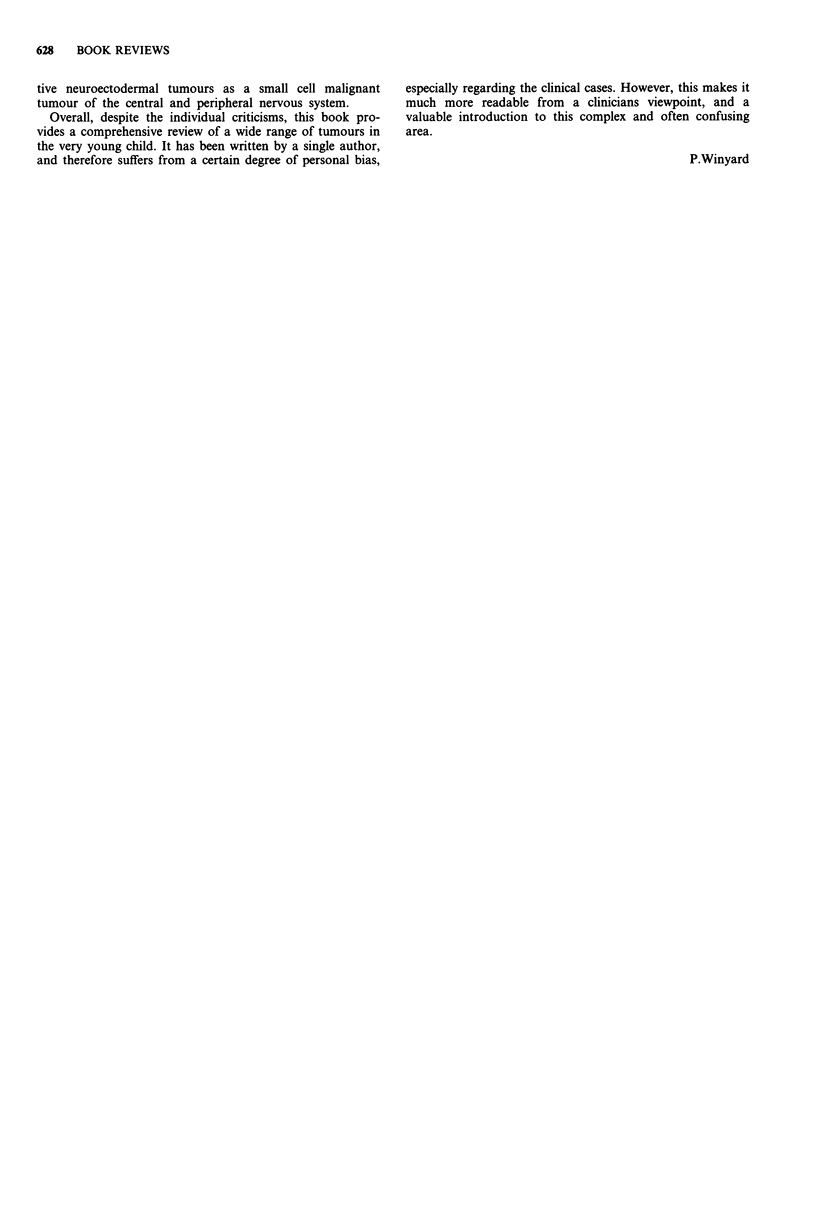# Tumours of the Newborn and Infant

**Published:** 1992-04

**Authors:** P. Winyard


**Tumours of the Newborn and Infant,**
H. Isaacs, Jnr., St. Louis: Mosby-Year Book Inc., 1991, 251 pp. £53.50.

This book represents an attempt by Hart Isaacs Jnr to demonstrate the 'gross pathologic and histopathologic features of neoplastic diseases and tumour-like conditions during the first year of life'. It is based largely upon the author's own experience over the last 27 years at the Children's Hospital of Los Angeles, with cross-references to studies from other centres.

The primary target for the publication is pathologists, with the specific aim being to help them interpret difficult histopathological appearances in developing, rather than mature tissues. However, it does include numerous case histories which increases its relevance to clinicians.

The first four chapters present a general background to tumours in infancy including aetiology, incidence/mortality, and diagnositic considerations. These are fairly succinct, although the incidence statistics could be clarified by using more uniform populations and comparable definitions. However, I must disagree with the author's unequivocal view that needle biopsy is contra-indicated in the initial diagnosis of paediatric solid neoplasm – 'since it is rare for enough tissue to be obtained and it is possible to miss the diagnostically important area' (Plus 'formal exploratory operations have fewer serious immediate complications'). This view may have been sound many years ago, but CT and ultrasound guided biopsy is now so accurate, and so much less invasive than open biospy, that needle biopsy increasingly has a role in tissue diagnosis.

The subsequent sections on specific disease cover a wide range of material from specific organ-related tumours, such as renal and liver tumours through to more nebulous concepts such as soft tissue tumours and tumour-like conditions of the skin. Most of these are clearly written with excellent photographs, especially of the gross pathological specimens. Unfortunately some of the photomicrographs lose their impact by being printed in black and white, especially when the arrow markers are in black too.

The chapters on retinoblastoma and renal tumours are particularly notable for their clarity and depth, respectively. However, I fail to see how the section on neuroblastoma could include so little detail about stage 4 S neuroblastoma, which is by definition a 'tumour of infancy' (Also, no mention of deletion of the short arm of chromosome 1 as a prognostic indicator). Another contentious area deserving greater attention, is the supposed 'unifying concept' of primitive neuroectodermal tumours as a small cell malignant tumour of the central and peripheral nervous system.

Overall, despite the individual criticisms, this book provides a comprehensive review of a wide range of tumours in the very young child. It has been written by a single author, and therefore suffers from a certain degree of personal bias, especially regarding the clinical cases. However, this makes it much more readable from a clinicians viewpoint, and a valuable introduction to this complex and often confusing area.

P.Winyard